# Infectious disease-related laws: prevention and control measures

**DOI:** 10.4178/epih.e2017033

**Published:** 2017-07-25

**Authors:** Mijeong Park

**Affiliations:** Office of Policy Development for Healthy Society, College of Medicine, Seoul National University, Seoul, Korea

**Keywords:** Infectious disease prevention and control, Middle East Respiratory Syndrome, Epidemiology, Human rights, Governance

## Abstract

**OBJECTIVES:**

This study examines recently revised Korean government legislation addressing global infectious disease control for public health emergency situations, with the aim of proposing more rational, effective and realistic interpretations and applications for improvement of law.

**METHODS:**

The Korea reported its first laboratory-confirmed case of Middle East Respiratory Syndrome (MERS) coronavirus on May 20, 2015. Since the first indexed case, Korean public health authorities enforced many public health measures that were not authorized in the law; the scope of the current law was too limited to cover MERS. Korea has three levels of government: the central government, special self-governing provinces, and si/gun/gu. Unfortunately, the Infectious Disease Control and Prevention Act does not designate the specific roles of each level of government, and does not state how these governmental branches should be vertically integrated in a state of emergency.

**RESULTS:**

When thinking about these policy questions, we should be especially concerned about introducing a new act that deals with all matters relevant to emerging infectious diseases. The aim would be to develop a structure that specifies the roles of each level of government, and facilitates the close collaboration among them, then enacting this in law for the prevention and response of infectious disease.

**CONCLUSIONS:**

To address this problem, after analyzing the national healthcare infrastructure along with the characteristics of emerging infectious diseases, we propose the revision of the relevant law(s) in terms of governance aspects, emergency medical countermeasure aspects, and the human rights aspect.

## INTRODUCTION

The Korean positive law at the time of the first confirmed case of Middle East Respiratory Syndrome (MERS) in the Republic of Korea (hereafter Korea) in 2015 had ambiguity in the legal description of infectious diseases and actors, which caused issues in the infectious disease response system led by the Korea Centers for Disease Control and Prevention (KCDC), including problems with infection control in medical institutions, with disclosure of patient information, and lacked directives regarding loss compensation. After that first case of MERS subsided, the law was revised, which resulted in reinforcement of the leading and commanding power of KCDC, increases in the number of epidemiology investigators, and establishment of standards for infection management in medical institutions and facilities. In addition, a legal basis to compensate for loss of patients with infectious diseases and fatalities was also prepared (Act No. 14316, Infectious Disease Control and Prevention Act).

There are multiple laws on infectious diseases in the Korea, including the Infectious Disease Control and Prevention Act. The laws are diverse: a) the Framework Act on the Management of Disasters and Safety, which is the foundation to take necessary measures and comprehensively mediate all works related to disasters occurring in the relevant administrative area; b) the Act on the Protection, Use, etc. of Location Information that is related to requests for information; c) the Police Act on collaboration for isolation and surveillance; d) the Regional Public Health Act for collaboration between community health centers affiliated with local governments; e) the Pharmaceutical Affairs Act that is related to emergency medical countermeasures; f) the Medical Service Act that is related to containment of medical institutions; and g) the Emergency Medical Service Act regarding quarantine facilities. These diverse laws should have a consistent form to help prevent and control infectious diseases.

## MATERIALS AND METHODS

**Figure 1.**
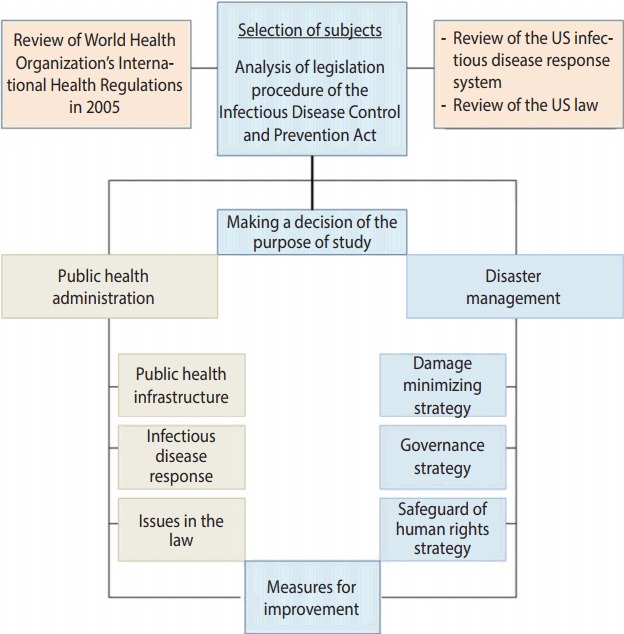
Framework of the study.

Due to the diversity of the infectious disease related laws, the laws need to be more systematic and consistent with each other, reflecting the inclusive and multifaceted characteristics of the public health area and the national health care system. Measures for response to emerging infectious diseases require collaborative governance that distinguishes the role of the central government from those of metropolitan cities/provinces and si/gun/gu, and then closely interconnects them. In addition, it should protect the human rights and privacy of those who are exposed to infectious diseases, and be in a form ensuring commonly shared rights of all the subjects. In the present paper, we reviewed the legislative procedure of the Infectious Disease Control and Prevention Act, analyzed its problems, and then proposed the direction to improve the Infectious Disease Control and Prevention Act, in part by modeling the National Incident Management System of the US (Figure 1).

## RESULTS

### Roles of infectious disease-related laws

Prevention and control of infectious diseases aims to prevent avoidable risks, to foster early detection of all risks, and to respond rapidly and effectively [1]. The laws have established direct foundations for spread control based on scientific evidences from the surveillance, notification and report of infectious diseases, the supply of hospital treatment and emergency medicine to minimize damage, and financial support for recovery. The actors for these roles are the central government, local governments, and medical personnel, who play the roles within the infrastructure comprising the national health care system, including development of health care resources, organization of the resources, health care management, supply of health care services, and financial supports [2]. Components of infrastructure in the national health care system seek to prevent pandemics of emerging infectious diseases. In cases of emerging infectious diseases where vaccines or drugs are insufficient or unavailable, the development and organization of resources, the supply of health care services, and financial support all have significant effects on response to infectious diseases and minimization of damage [3]. For example, the indicated domestic causes of MERS were individual problems of vulnerabilities of the national infectious disease management system and nosocomial infection prevention control, inefficiency of health care delivery systems, health care use and nursing culture, as well as correlations between the problems [4].

Since the 2000s, international organizations such as the World Health Organization (WHO), the International Food and Agriculture Organization, and the World Organization for Animal Health (Office International des epizooties) have actively promoted international collaboration to respond to uncertainty and mutation potential of pathogens and to prevent spread of emerging infectious diseases. These are also closely related with the reason national health care systems made efforts to establish better infrastructure. These international organizations announced an international common strategy for control of avian influenza through the Beijing Declaration in 2006, by which they asked for development of an integrated action plan for each country and practical performances [5].

In 2005, the WHO revised the International Health Regulation (IHR) (prepared by treaty in 1969), expanding its scope to infectious disease control, protection of human rights, fair trade, environmental protection, and security [6]. In particular, IHR 2005 recommended collaboration of the international community to prevent and respond to incidents and spread of infectious diseases. In addition, IHR 2005 introduced the concept, ‘public health emergency of international concern’ [7]. Since then, emerging infectious diseases that did not exist previously and recurring infectious diseases have emerged or spread before acquiring herd immunity. This has been found all over the world including Africa, Southeast Asia, South America, Europe, and the US and the concept expanded the scope of response from containment of infectious diseases within the border to a supranational level [8].

IHR 2005 also affected the law of the Korea: the ‘Communicable Diseases Prevention Act.’ On December 29, 2009, the Communicable Diseases Prevention Act was fully revised (enforced on December 30, 2010), and the term, ‘communicable diseases’ has been changed to ‘infectious diseases’ so as to include both communicable and non-communicable diseases. The revision was not limited to the term. In Aticles 2, 11-13 of the Infectious Disease Control and Prevention Act, Class 1-5 infectious diseases and designated infectious diseases were named legal infectious diseases, because they were designated by law. On the other hand, WHO monitors infectious diseases, bioterror infectious diseases, sexually transmitted infectious diseases, zoonotic infectious diseases, and health care-related infectious diseases, and these were designated as notifiable infectious diseases by the Minister of Health and Welfare (MOHW).

**Figure 2.**
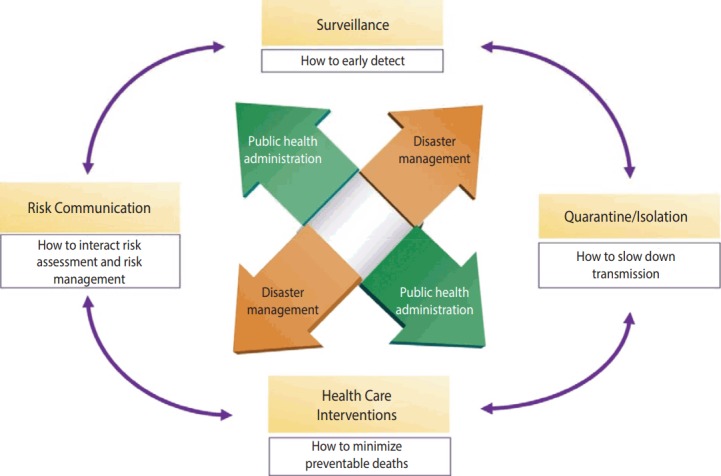
Flow chart of infectious disease response measures.

Measures of response to infectious diseases can be summarized as Early Detection (surveillance) - Non-pharmacological Intervention (quarantine/isolation) - Medical Intervention (health care intervention) - Risk Communication, in which patients with infectious diseases are early detected by surveillance of infectious diseases, and transmission is maximally prevented through nonpharmacological intervention such as quarantine and isolation, damage is minimized through vaccination and drug distribution, and risk communication is conducted based on risk assessment. All of these activities are fulfilled through public health administration and disaster management (Figure 2).

Public health administration is to apply technologies and principles of health care for health of populations based on all knowledge available in the areas of medicine and public health [9]. The WHO defined the scope of public health administration as 1) preservation of public health records, 2) health education to the public, 3) environmental sanitation, 4) infectious disease control, 5) mother and child health, 6) health care, and 7) nursing and statistics of public health [10].

According to Article 3, Clauses 1-3 of the Framework Act on the Management of Disasters and Safety, damages by infectious diseases are considered as a kind of social disaster. Thus, when infectious diseases break out, a command system for disaster management, unlike normal administrative management, in collaboration with public health experts, is required. The scope of disaster management includes advanced prevention, minimization and recovery activities of human and material damage, a supply of a minimum level of convenience for a certain period of time, and then return to daily life after risk situation is ended.

National intervention that is in the form of public health administration and disaster management are performed through management support systems and command response systems. A management support system is a risk response plan that mediates response strategies by securing comprehensive information about the incident, and makes a decision for a response level and required support ranges of human and material resources. A command response system is composed of authorities to control and command, which are required for response measures in the field, so that command response system can be different depending on infectious disease, and local governments or medical institutions may have their own command response systems.

The concepts of the management support system and the command response system can be found in the National Incident Management System that was standardized by the Centers for Disease Control and Prevention (CDC) of the US, in which ‘Incident Management System’ and ‘Incident Command System’ have been established in relation to public health risk response by infectious diseases in order to facilitate mediations between all levels of governments, including the Federal Government, state governments and local (city and county) governments, plus public, private and non-profit organizations [11]. The Incident Management System classifies potential risk factors for public health into biological, artificial, intentional, and natural factors, determines the order of risk management based on seriousness of all potential risk factors for public health, chance of incidence, and response ability, and then takes appropriate measures as a response [12]. The US CDC supports state governments and the Department of Health and Human Service. The Incident Command System refers to a temporary management procedure that can manage finance, human resources, facilities and risk communication from the time of incidence. The Minister of Health and Human Service (HHS) forms the HHS Secretary’s Command Center, in which he/she makes preparations at all health care levels, and performs response and recovery activities. However, an incident manager is selected at the incident field, who operates an emergency organization that flexibly responds depending on type and severity of the incident, and all staff persons participating in the incident response report only to the incident manager.

The importance of the command response system and the management support system is related to the matter of governance. When the first MERS patient was confirmed in the Korea on May 20, 2015, the crisis stage of infectious diseases was ‘Caution’ stage. Nevertheless, the type of emergency organization was continuously changed, from KCDC (Countermeasure Headquarter) to the MOHW (Central MERS Management Headquarter), to the Ministry of Public Safety and Security (Pan Government MERS Countermeasure Support Center), and then ultimately to the Blue House (MERS Emergency Response Team). On June 4, ‘Comprehensive Public-Private Response Task Force for MERS’ led the response. On June 8, ‘Immediate response TF’ mainly composed of private experts was implemented, which was conferred with full authority for nosocomial infection management and command of administrative support. On June 9, the ‘MERS Immediate Response Team’ was organized. Even the commanding authority was transferred from the spokesperson of the Blue House to the acting prime minister, to the Chief Secretary of Policy Coordination and to the President. As such, there were problems in the command response system [13].

While the management support system can be guided by a manual, there was no consistency in manual instructions after the MERS outbreak, which intensified the confusion. A manual is written to assess risks of infectious diseases in advance, whereas it can be added with confirmation of specific activities or selection of essential measures after outbreak of infectious diseases. Nevertheless, it is important for the government to apply the manual to the field without confusion even after modification.

### Infectious Disease Control and Prevention Act (Act No. 14316)

#### Legislation procedure after outbreak

The second largest MERS outbreak in the world (after Saudi Arabia) occurred in the Korea, in which MERS was not initially considered as a legal infectious disease when patients were confirmed. Although Article 2 of the Act delegated Class 4 infectious diseases to the enforcement rule (i.e. the decree from the MOHW), MERS was not included in this enforcement rule, but labeled as an emerging infectious disease syndrome only in the criteria for diagnosis and declaration of legal infectious diseases. Thereafter, the MOHW designated it as a Class 4 infectious diseases after revision, in which the disease was officially named as MERS, being considered as an infectious disease that newly occurred or may occur in the country or as an international epidemic infectious disease that has a chance to be transmitted to the entire country.

According to the report on MERS prevention and response after an inspection conducted by the Board of Audit and Inspection of Korea, problems in the early response were delayed implementation of an epidemiological investigation in spite of the declaration by the patient, and early termination of the epidemiological investigation even without considering the chance of secondary infection within the medical institutions [14]. In addition, the medical institutions were found to have issues, including overcrowding in emergency centers of general hospitals, settings of emergency centers operated as multi-patient rooms, and increased chance of infection due to family nursing [15]. Thus, the Infectious Disease Control and Prevention Act was revised, focusing on reform of infectious disease control system, supplementation of specialists in infectious diseases, preparation of measures to manage and oversee infection in hospitals, improvement of structure and culture of emergency centers, compensation of medical institutions and pharmacies for loss, compensation of patients and fatalities, and requests and disclosure of information at the time of any public health crisis caused by infectious diseases. However, there have been ongoing discussions about governance issues for collaboration between medical institutions and community health centers that are critical for early response; hospital response issues such as sufficient isolation facilities; and classification system of legal infectious diseases [16].

#### Issues of the Infectious Disease Control and Prevention Act Aspect of policy and management

A more transparent decision-making and rapid response for prevention and control of infectious diseases can be enabled by clearly defining the legal subject and scope. However, public health risk factors have been increasing in random and atypical forms, so that it has become harder to accurately assess their influences and severities, classify infectious diseases and define their scopes. In addition to disease onset evidences based on incidence rate, mortality rate and epidemiological information, chances of local incidence or inflow from other countries, chance of prevention and treatment, and sensitivity of the public to diseases, there are more diverse evaluation criteria. To evaluate potential risks caused by influenza virus, the US CDC has proposed assessment criteria through the Influenza Risk Assessment Tool, in which viral characteristics, population characteristics, and ecological and epidemiological standards were presented [17]. To assess risk of seasonal influenza, the European Union conducts survey in the countries that experience influenza every year, in addition to collecting clinical, epidemiological and virological data, by which seriousness of infectious diseases is evaluated [18]. The Tool for Influenza Pandemic Risk Assessment in the WHO uses influences, risk of transmission, and necessity to ban travel or trade as assessment criteria [19].

Classification of infectious diseases is related to a declaration system, because the time to declare and report to community health centers and KCDC is different depending on the infectious disease. According to the report by the Board of Audit and Inspection on requests for medical care benefits from 1,499 medical institutions in Seoul between October 2015 and September 2016, 1,221 (81.5%), institutions failed to properly report incidences of chickenpox. In addition, 656 (79.6%) institutions in 824 medical institutions failed to report mumps [20].

In Article 2, Clause 8-12 of the Act, the legal subject is infectious diseases notified by the MOHW, leading to understanding infectious diseases as an administrative measure with general and abstract characteristics, so that it may be unable to conduct rapid diagnosis and report in a timely manner [21]. Regarding the legal aspects, notification is an administrative measure that an administrative agency publicly announces to unspecified individuals, only playing a role of spreading information, whereas notification itself has no legal effect related to the people’s rights and duties. To become a legal order, notification is required to have specific and clear legal delegations [22]. The requirement of specific description about each individual article as a foundation of delegation is to protect the people’s basic rights. Considering the characteristics of response measure to infectious diseases that may cause conflicts between the duty of the state to protect the people’s basic rights and the individuals’ rights to move freely, notification has a limitation in protection of basic rights. In addition, notification as a specific norm control becomes effective five days after a public announcement unless there are special regulations in other laws and announcing documents [23]. This may be contradictory to the immediate compulsory provisions, such as isolation and quarantine that can be performed in the field.

### Aspect of organization of medical resources and delivery of health care service

The principal personals of measures of response to infectious diseases are medical professional who report the first suspected patients. Medical personnel must report to the head of the affiliated medical institution or to the head of the relevant community health center if not affiliated with a medical institution (Article 11 of the Act). According to Article 18 of the Act, medical personnel or the head of a medical institution can request the MOHW to implement an epidemiological investigation. The purpose of a request for epidemiological investigation is to rapidly conduct an epidemiological investigation when disease onset has occurred or when it is suspected to have a chance of incident. Nevertheless, this Article can be restrictively interpreted to mean that only medical personnel who diagnosed the patients are allowed to request for an epidemiological investigation. If it is necessary to restrict the eligibility to request epidemiological investigation, it should be revised to ‘medical personnel who had treated patients with infectious diseases etc. or with being concerned to have infectious diseases etc.’

Article 42 of the Act is for compulsory disposition for infectious diseases, in which the subject of the right is the MOHW, a mayor/governor of province, or the head of si/gun/gu. Although this gives authority to enforce inspection, diagnosis, treatment or hospitalization at the site where patients suspected to have infectious diseases are present, they have been stated in parallel, making it confusing to identify which decision maker has priority. Since epidemiology investigators from metropolitan cities/provinces and community health centers can be dispatched to the field with the same authorities, it seems that confusion in the field could be unavoidable. According to Article 47 of the Act, epidemiology investigator has the authority to temporarily close the relevant site when infectious diseases occur. Although its intention is to allow the epidemiology investigator to take a measure for control of infectious diseases in emergency, it is vague what kind of administrative activities can be performed by the authority to temporarily close the relevant site. Per Article 60 of the Act, the similar authority to command in the field belongs to an epidemic control commissioner. An epidemic control commissioner is appointed either among public officials of metropolitan cities/ provinces or among those in the MOHW. Since he/she has the authority to take response measures at the incident site of infectious disease, there can be conflicts of duties between the epidemic control commissioner and the epidemiology investigator in the field. It would be desirable to stipulate the scopes and details of duties for epidemic control commissioners and epidemiology investigators in the Act in order to clearly describe what measures can be taken in what situation.

If response at the outbreak needs to be considered as an activity of disaster management, it should be consistent with the Framework Act on the Management of Disasters and Safety. Duties of Grade 4 to 9 national public officials and local public officials who work for emergency safety inspection per Article 30 of the ‘Framework Act on the Management of Disasters and Safety’ are stipulated in the ‘Act on the Persons Performing the Duties of Judicial Police Officers and the Scope of Their Duties.’ The Act also has stipulated duties of public officials in special metropolitan city, metropolitan city, special self-governing city, province, special self-governing province and si/gun/gu. Since the authority of an epidemic control commissioner to control in the field is a compulsory power, the duty of public health response should be included in the scope of this duty.

### Aspect of organized arrangement of resources and financial support

Quarantine, isolation and duties of emergency organization implemented following a crisis alert level by infectious diseases are different depending on the risk of infectious diseases. High risk infectious diseases are controlled by KCDC. In contrast, metropolitan cities/provinces and si/gun/gu respond to low risk infectious diseases. Thus, the leading organization and actor can be different depending on the site of an infectious disease incident, the laboratory/examining room, the designated medical institution for treatment and isolation, and quarantine/isolation protocols in place. Therefore, the definition and scope of public health administration for implementing emergency organization should be stipulated, and the authority organization and commanding officer need to be stated in parallel in order to make the necessary level of national intervention for infectious disease be proportional to its risk. If not stipulated in the Act, governance to mediate duties of public officials who are in charge of public health practice is required, focusing on more closely related duties in order to less interfere with each other. For example, when isolation is requested by a Quarantine Station to KCDC and also by community health centers to metropolitan cities/provinces, nationally-designated beds are assigned for inpatient treatment. In case of a priority issue in this process, there should be a matching management system.

To use emergency medicines, approval, review and cross certification of drugs is required due to the Free Trade Agreement in the drug area, so that the domestic law reflects the standards for drug qualities, non-clinical trials and clinical trials meet the international standards based on understanding of approval and review systems of each country [24], because approval of drug products can be delayed or declined due to the difference in data that are required for import of drugs developed in foreign countries.

In the US, the ‘Pandemic and All-Hazards Preparedness Act 2006’ was legislated to improve the legal system, by which the right to use Food and Drug Administration (FDA)-unapproved drugs in emergency was strengthened [25]. ‘Pandemic and All-Hazards Preparedness Reauthorization Act 2013’ regulated the right to use unapproved drugs in emergency [26]. The ‘21st Century Cures Act’ extended the range of clinical evidences required for FDA approval, and waived the requirement of clinical trial in some cases, which was passed unanimously by common consent of the relevant committee in the US House of Representatives in 2015 in order to promote development and rapid approval of new drugs and medical devices [27]. However, this law could lead to the approval of drugs and medical devices that are below the current standards of safety and efficacy [28]. Therefore, it is important to prepare rapid evaluation criteria for to follow-up on any drugs receiving rapid approval in crises, in order to prevent undesirable consequences in patients and the health care system.

## DISCUSSION

Since the conditions for outbreaks of infectious diseases keep changing, potential epidemics can be difficult to recognize early. In addition, the conditions are complex and sometimes contradictory. Thus, preparation for infectious diseases and response measures can be a ‘nasty and abstruse dilemma’ that a country is unable to achieve on her own [29]. The law for response to various risks to public health, including increasing emerging infectious diseases with unknown causes, deliberate terrorism, and various big accidents in cities, should build a stable foundation for national intervention without invading human rights. All humans’ dignity should be respected, and their dignity should be universally guaranteed under the rubric of basic rights, which should be common benefits.

To reduce social prejudice and discrimination towards human immunodeficiency virus/acquired immune deficiency syndrome (AIDS), ‘the 3rd National Health Plan 2011-2020’ was established in the Korea, aiming at improvement of discriminatory attitudes toward AIDS patients from 44.6% in 2008 to 15.0% in 2020 [30]. We anticipate a serious pursuit of the laws and governance required for control and response to infectious diseases, not only by focusing on the rights guaranteed by the Constitution, but also by understanding human rights and human dignity as universal values.

### Directions of revision for infectious disease related laws

The Infectious Disease Control and Prevention Act functions as a Special Act to lay foundations for epidemiological investigation, quarantine, isolation, laboratory diagnosis, vaccination and treatment. At the same time, the Act should function as a Framework Act to control and treat infectious diseases as social disasters. It is anticipated for infectious disease related laws to be framework acts that protect the people’s life, body, health and property through interventions by the central government and local governments per Articles 34-36 of the Constitution of the Korea, while reflecting the specialties of control and response measure for infectious diseases.

#### Governance

Since an international legal strategy is required for formation of governance to control emerging infectious diseases [31], its scope of application should be within the level reflecting the major changes in IHR 2005. While MERS outbreaks in Korea began as a local infectious disease, it came to be considered as a national disaster, leading to the implementation of the Central Disaster and Safety Countermeasures Headquarters, which demonstrates the importance of early field response step after outbreaks of emerging infectious disease. However, there could have been a more rapid and effective disaster management if local governments had authority and performance. A system should be established to confer the practical legal authority to a local government close to the region of incident for appropriate functions, enabling them to take a field response proper for the crisis situation, once determined serious.

Disaster management systems by local governments, a collaborative system between related agencies, and field command systems should be stipulated in the Act. While the leading agency for disaster management should be a central administrative agency, local governments can be agencies in charge of local disaster management. Expenses and final responsibilities may be charged to different subjects depending on the characteristics of administration. The responsible agency will differ depending on the type of disaster, but it is desirable to distinguish scope of responsibility depending on metropolitan self-governing government and basic self-governing government. In addition, local governments need to prepare ordinances to judge if administrative duties regulated to be performed by the head of local government are relevant to delegation duties of the agency, based on which they can systematically collaborate. In addition, a delegation provision should be added in order to request or command mutual supports between local governments and administrative helps by classifying crisis situations depending on the cause, either by infectious disease or by other risk factors.

Emergency organization that is implemented in public health crisis is different depending on each crisis stage, and response measures by related local governments can be adjusted accordingly. The law has no specific descriptions, and role assignments for exceptional situations have been selectively stated in the manual. The Framework Act on the Management of Disasters and Safety regulated that the head of si/gun/gu must write a ‘Field Manual for infectious diseases’ containing instructions for prevention, preparation, response, and restoration of disasters as well as other required items. To request for collaboration from two or more metropolitan cities or provinces, the range of damage should be so severe that it is determined to be a social disaster. However, if there is no damage across multiple metropolitan cities or provinces, they may not need to collaborate. Therefore, the manual should be summarized in consistency by adjusting the scale of collaboration based on the range of incidence.

#### Emergency medical countermeasure

For emergency use of any drug that had neither approval nor declaration, at least three obligations should be stipulated in the law. These obligations include preparing a measure to control safety of patients for using drugs approved only for emergency, developing a standard procedure for emergency use of drugs, and a meeting of the appropriate officials to determine emergency use of drugs depending on the public health crisis.

To prepare a measure to control safety of patients for using drugs approved only for emergency, an emergency use approval system should be prepared. Specific approval procedures need to be established for any case when relevant drugs are used for indications different from their approved uses, and also for cases when the drug has never been approved before. Currently, the Pharmaceutical Affairs Act and the Medical Devices Act order that drugs and medical devices must obtain approval or report before commercialization in order to verify safety and efficacy [32]. To rapidly verify and approve use of drugs without these procedures, a separate procedure, with general approval, is needed. In the US, an ‘Animal Rule’ has been applied to approve drugs with assumption of clinical benefits based on results of animal experiments [33]. According to the Animal Rule, it is important to develop a data list to judge the approval of a drug in an emergency, which includes a stated purpose and use of the subject drug, medical necessity, approval status, safety and efficacy information, manufacture and quality information, risk and benefit assessment data, and description of any drug-related facts. To let the MOHW decide items such as expiration day, scope and condition for drugs approved for emergency use, KCDC and the Ministry of Food and Drug Safety should share such information and discuss this even before outbreaks of emerging infectious diseases.

#### Safeguard for human rights

##### Improvement of subject range for epidemiological investigation

To secure legitimacy of compulsory disposition by public officials during an epidemic of infectious diseases, subject and method for compulsory measures and its performance procedures need to be improved, leading to practice of principles of the minimum infringement and proportionality [34]. No matter what benefits are gained or lost by the limitation of basic rights, basic rights are a universal and essential value, so that they should be guaranteed during the pursuit of any compulsory measure.

Clear distinction of subjects for epidemiological investigation is related to the limitation of national intervention by the principle of the minimum infringement. Article 18, Clause 3 of the Act defines subject of epidemiological investigation as ‘anyone.’ However, subjects of epidemiological investigation in Article 41 of the Act are patients with infectious diseases. From the medical perspective, patients with infectious diseases refer to ‘patients with infectious diseases, patients with infectious disease-like, and pathogen carriers.’ However, they are considered as subjects of the same administrative order for treatment, while the people’s basic rights might be ignored.

Epidemiological investigation aims to identify or speculate how confirmed patients were infected, and to assess the potential for additional confirmation of patients who were closely contacted with each confirmed patient previously. On the other hand, subjects of epidemiological investigation can be classified into high risk group, intermediated risk group, low risk group, and no risk group depending on the risk criteria for infection by the infectious disease in question. Each group from such classifications can be divided into a symptomatic group and an asymptomatic group [35]. Quarantine and isolation for asymptomatic group means limitation of free movement; the range and condition for limitation of free movement should be proposed based on scientific evidence.

Article 42 of the Act is about compulsory disposition for patients with infectious diseases. Patients with infectious diseases corresponding to specific infectious diseases can be forced by law to have inspection and diagnosis. If the diagnosis resulted in confirmation of infectious diseases, the patients can be forced to be hospitalized. Compulsory disposition for patients with infectious diseases are distinct from the duty of inpatient treatment in Article 41. Compulsory diagnosis or compulsory hospitalization are kinds of immediate compulsion in terms of administration, which is practiced ‘when administrative damage occurs, when occurrence of disability is about to occur, when request of individual to fulfill the duty is unable to attain the goal or when there is no sufficient time to request to fulfill the duty [36].’ To justify immediate administrative compulsion, it needs to be carefully practiced considering its potential to violate the people’s basic rights. In addition, methods less invasive of basic rights than immediate compulsion must be applied first [37]. Therefore, regulations should be specified including specific condition and procedure for compulsory disposition, isolation period, discharge condition, and opportunity to describe opinion [38].

##### Improvements of the Prevention of Acquired Immune Deficiency Syndrome Prevention Act and the Tuberculosis Prevention Act

In 2009, the ‘Communicable Disease Prevention Act (Act No. 308)’ was completely revised, resulting in the Infectious Disease Control and Prevention Act, and since then, the term, communicable disease, is not used any more. In addition, the Parasitic Disease Prevention Act was repealed in the same year. On the other hand, the Tuberculosis Prevention Act enacted in January 1967 and the Prevention of AIDS Prevention Act enacted in November 1987 are still in place [39]. It should be empirically reviewed if such independent regulations with disease names result in the restriction of human rights of patients with corresponding infectious diseases continuously and substantially.

At first, it should be investigated if there is any reasonable reason to have different duties and punishments between the Infectious Disease Control and Prevention Act and those two Acts. In the Infectious Disease Control and Prevention Act, all declining activities by subject of epidemiological investigation, e.g., a false report, submission of false data, intentional omission or concealment of truth, are to be punished by ‘imprisonment’ for not more than two years, or by a fine not exceeding 20 million Korean won (KRW) (Article 79). In contrast, violation of rules for hospitalization or home isolation for patients with infectious diseases is punished by a fine not exceeding 10 million KRW. In addition, a false statement to medical personnel in disasters (Article 35) is punishable by a fine not exceeding 10 million KRW. In the Prevention of AIDS Prevention Act, a person who performs an act of carrying and spreading AIDS are punished by imprisonment for not more than three years (Article 25 of the Act), imprisonment for not more than one year, or a fine not exceeding 3 million KRW (Articles 10 and 27, Clause 2). The Tuberculosis Prevention Act has non-punishment regulation [40].

In emphasizing the importance of epidemiological investigation, it is reasonable to separate the regulations by punishment rule for spread of infectious diseases through rejection of epidemiological investigation and non-punishment rule for simple rejection of epidemiological investigation. In some cases, failure to get collaboration for epidemiological investigation leads to abrupt risk for disease spread, and probability of damage, which brings the person to account. In some cases, on the other hand, there is no clear and absolute evidence of risk for public health even without compulsory isolation such as in asymptomatic suspected patients. If separation of subjects for duty is medically valid, it would be reasonable to improve provisions for fair punishments.

If separation is not reasonable, subjects of epidemiological investigation should be unified the same ranges. In the Prevention of AIDS Prevention Act and the Tuberculosis Prevention Act, subjects of epidemiological investigation are defined as ‘the infected or those who have sufficient reasons to be suspected for infection.’ If persons having duty to cooperate with epidemiological investigation were defined in consideration of these two infectious diseases for separate compulsions even without specific descriptions about the meaning of ‘sufficient reasons,’ it could be a violation of principles in the balance of legal interests and suitability of means. Benefits of basic rights are universal, so the reason to restrict universal basic rights should be based on a strong justification appropriate for its subject [41].
