# Supplementary material for: Infectious disease-related laws: prevention and control measures
Source: Epidemiol Health. 2017 Jul 25;39:e2017033. doi: 10.4178/epih.e2017033 (PMC5675986; doi:10.4178/epih.e2017033)
Supplement: Supplementary file 1 [file epih-39-e2017033-supplementary.pdf]

Volume: 39, Article ID: e2017033

https://doi.org/10.4178/epih.e2017033

감염병 관련 법률의 문제점과 개선 방안

박미정

서울대학교 의과대학 건강사회정책실

Infectious disease-related laws: prevention and control measures

Mijeong Park

Office of Policy Development for Healthy Society, College of Medicine, Seoul National University, Seoul, Korea

Corresponding author: Mijeong PARK

E-mail: apotre@snu.ac.kr

Volume: 39, Article ID: e2017033

https://doi.org/10.4178/epih.e2017033

초 록

본 연구의 목적은 2015년 메르스 발생 후, 감염병의 예방 및 관리에 관한 법률의 입법경과를 검토하여 감염병으로 인한 공중보건위기 대응과 관련된 법률의 보다 구체적인 개선방안을 제시하는 것이다. 중앙정부와 지자체의 협력을 위한 거버넌스, 응급상황에서의 긴급의약품 제공을 위한 절차, 격리 대상자의 인권보장과 관련된 부분의 충분한 법적 상당성을 갖추기 위해 국가 보건의료체계 하부구조 측면에서 문제점을 분석한 후 법률 개정방안을 제언하였다.

찾아보기 말: 감염병의 예방 및 관리에 관한 법률, 감염병 대응, 메르스, 인권, 역학조사, 거버넌스

Volume: 39, Article ID: e2017033

https://doi.org/10.4178/epih.e2017033

I. 서론

2015년 한국에서 메르스가 발생할 당시의 실정법은 법정 감염병의 대상과 행위주체의 모호함으로 인하여 질병관리본부를 중심으로 한 감염병 대응체계, 의료기관의 감염관리, 정보공개와 손실보상 등에서 문제점을 나타냈다. 메르스가 진정되면서 법률개정을 통하여 질병관리본부의 지휘·통제권이 강화되었고, 역학조사관이 증원되었고, 의료기관의 감염관리와 시설의 기준이 마련되었다. 그리고 감염병 환자와 사망자에 대한 손실보상에 관한 법적 근거도 마련되었다(감염병의 예방 및 관리에 관한 법률 제14316호).

국내 감염병 관련 법률은 감염병의 예방 및 관리에 관한 법률을 비롯하여 다수가 있다. 해당 관할 구역에서 발생한 재난에 관한 사항을 총괄하여 조정하고 필요한 조치의 근거가 되는 재난 및 안전관리 기본법, 정보요청과 관련되는 위치정보의 보호 및 이용 등에 관한 법률, 격리와 모니터링 협조를 위한 경찰법, 지자체 소속의 보건소간 협력을 위한 지역보건법, 긴급치료와 관련되는 약사법, 의료기관 폐쇄와 관련되는 의료법, 격리시설과 관련되는 응급의료에 관한 법률 등 다양한 법률이 감염병 예방과 관리를 위해 일정한 형식을 갖추어야 한다.

II. 연구방법

감염병 관련 법률의 다양함은 공중보건영역의 포괄적이고 다면적인 특성이 국가 보건의료체

Volume: 39, Article ID: e2017033

https://doi.org/10.4178/epih.e2017033

계에 반영되어 관련 법규가 보다 체계적이고 상호 간의 정합성을 갖출 것을 요구한다. 신종 감염병 대응 조치는 중앙정부와 시·도, 시·군·구의 역할을 구별하여 긴밀하게 상호연결 되도록 하는 협력적인 거버넌스를 필요로 한다. 또한 감염병에 노출되는 사람들의 인권을 보호하고 모든 기본권 주체가 공통으로 향유하는 보장형식을 갖추어야 한다. 본 논문에서는 감염병의 예방 및 관리에 관한 법률의 입법경과를 고찰하여 문제점을 분석하고, 미국의 국가재난관리체계를 반영하여 감염병의 예방 및 관리에 관한 법률의 개선방향을 제언하였다 [Fig 1].

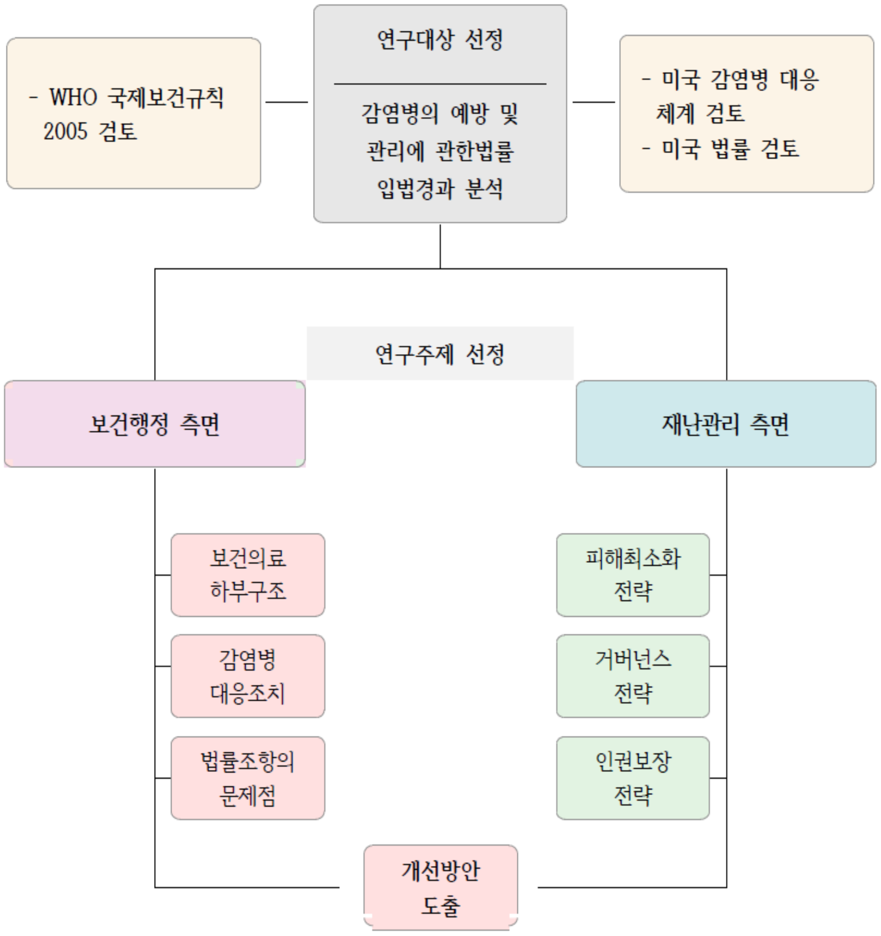

Fig 1. 연구의 틀

Volume: 39, Article ID: e2017033

https://doi.org/10.4178/epih.e2017033

III. 연구 결과

A. 감염병 관련 법규의 역할

감염병의 예방과 관리의 목표는 피할 수 있는 위협을 예방하고, 모든 위협을 조기에 감지하며, 신속하고 효과적으로 대응하는 것이다 [1]. 감염병 감시와 신고 및 보고를 기초로 과학적인 근거에 의한 확산 통제와 피해 최소화를 위한 병원 치료와 긴급 의약품 공급, 그리고 회복을 위한 재정지원의 직접적인 근거가 법규에 명시되어 있다. 국가와 지방자치단체, 그리고 의료인은 이러한 역할의 주체이다. 그리고 보건의료자원의 개발, 자원의 조직화, 보건의료관리, 보건의료서비스 제공, 재정지원 등 국가 보건의료체계를 구성하는 하부구조를 통하여 이 역할을 수행한다 [2]. 국가 보건의료체계의 하부구조 요소는 신종 감염병 대유행에 영향을 미친다. 예방백신이나 치료제가 없거나 부족한 신종 감염병인 경우 자원의 개발과 조직화, 보건의료서비스의 제공, 재정지원 문제는 감염병 대응과 피해최소화에 중대한 영향을 준다 [3]. 예컨대 메르스의 국내 발생 원인으로 국가방역체계와 병원감염 예방관리의 취약성, 의료전달체계 비효율성, 의료이용 및 간병문화 등 개별 문제점과 이들 문제점 간의 상호연관성이 지적되었다 [4].

2000년대 이후 병원체의 불확실성, 변형 가능성에 대응하고 신종 감염병의 확산을 막기 위하여 세계 보건기구(WHO), 국제 식량농업기구(FAO), 세계 동물보건기구(OIE) 등 관련 국제기구를 중심으로 본격적인 국제협력을 강화하는 이유도 국가 보건의료체계의 하부구조 마련과

Volume: 39, Article ID: e2017033

https://doi.org/10.4178/epih.e2017033

무관하지 않다. 이들 국제기구들은 2006년 베이징 선언을 통해 조류독감 관리에 관한 세계 공통의 전략을 발표하고, 각국의 통합된 국가 행동계획 개발과 실제적인 이행확보를 호소하고 있다 [5].

세계 보건기구는 1969년에 조약 방식으로 마련했던 국제보건규칙(International Health Regulation; 이하 IHR로 표기함)을 전염성 질병 통제, 인권보호, 공정무역, 환경보호, 안보 등 그 범위를 확대하여 2005년에 새롭게 개정하였다 [6]. IHR 2005의 특징은 감염병 발생 및 확산 예방과 대응을 위해 국제사회의 협력을 권고한 것이다. 또 '국제 관심사로서 공중보건 긴급사태(public health emergency of international concern)'라는 개념을 도입하였다 [7]. 과거에는 없었으나 새로이 질병을 일으키는 신종 감염병이나 집단면역이 아직 형성되어 있지 않은 재 출현 감염병이 아프리카, 동남아시아, 남미, 유럽, 미국 등 전 세계에서 발생하고 있기 때문에 국경에 감염병을 가두어 두었던 대응방안을 초국가적인 관점으로 확장한 대응 개념이라고 할 수 있다 [8].

IHR 2005의 권고는 '전염병 예방법'을 가지고 있었던 우리나라의 법률에 영향을 주었다. 2009년 12월 29일 전염병 예방법을 전부개정(2010.12.30.시행)하고, '전염병'이라는 용어도 전염성 질환과 비 전염성 질환을 포함하는 '감염병'으로 변경하였다. 용어만 변화된 것이 아니다. 감염병의 예방 및 관리에 관한 법률 제2조, 제11조-제13조에서 제1군-제5군 감염병과 지정 감염병은 법령으로 정하는 것이므로 법정 감염병이라 칭하고, 세계 보건기구 감시대상 감염병, 생물테러 감염병, 성매개 감염병, 인수(人獸)공통 감염병, 의료관련 감염병은 보건복지부 장관

https://doi.org/10.4178/epih.e2017033

의 고시 감염병으로 분류하였다.

감염병 대응 조치는 조기 감지(surveillance) – 비약물적 개입(quarantine/ isolation) – 의료적 개입(health care intervention) – 위기 소통(risk communication)으로 요약할 수 있다. 감염병 감시를 통해 감염병 환자를 조기에 찾아내고, 검역과 격리 등 비 약물적인 조치를 통해 최대한 확산을 방지하고, 예방접종과 치료제 배포 등을 통해 피해를 최소화하고, 위험성평가에 기반하여 위기 소통하는 이 모든 활동은 보건행정과 재난관리를 통해 이루어진다 [Fig 2].

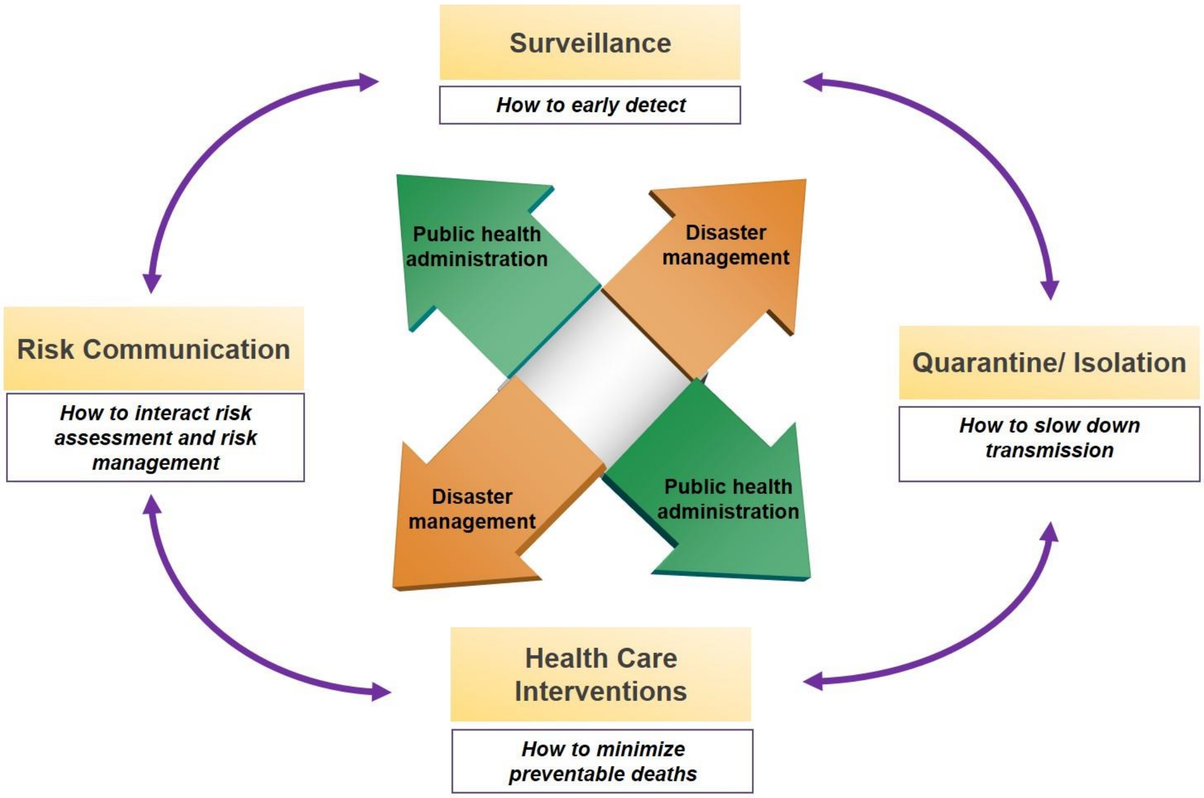

Fig 2. 감염병 대응 조치의 흐름도

보건행정은 의학과 공중보건학의 제반 지식을 근거로 인구집단의 건강을 위해 공중보건의

Volume: 39, Article ID: e2017033

https://doi.org/10.4178/epih.e2017033

기술과 원리를 응용하는 것이라고 할 수 있다 [9]. 세계 보건기구는 보건행정의 범위를 1)공중보건 기록의 보존, 2)대중에 대한 보건교육, 3)환경위생, 4)감염병 관리, 5)모자보건, 6)의료, 7)보건간호 및 보건통계로 정하고 있다 [10].

재난 및 안전관리기본법 제3조 제1호-제3호에 따르면 감염병으로 인한 피해는 사회재난의 일종으로 간주된다. 따라서 감염병이 발생하면 평상시의 행정관리와는 달리 공중보건 전문가와 협력하는 재난관리의 지휘체계가 필요하다. 재난관리의 범위는 사전예방, 인적•물적 피해의 최소화와 복구활동, 일정한 기간 동안 최소한의 생활편의 제공, 위험상황이 종료 된 후 일상생활의 복귀까지 포함된다.

보건행정과 재난관리라는 두 가지 형태의 국가개입은 관리지원체계와 명령대응체계를 통해 수행된다. 관리지원체계는 사건(Incident)전반에 대한 정보를 확보해서 대응전략을 조정하고, 대응수준과 인적•물적 자원의 필요한 지원범위를 결정하는 위기대응 계획이라고 할 수 있다. 명령대응체계는 발생현장에서 대응 조치에 필요한 통제권과 지휘권이 그 요소다. 그래서 감염병마다 다를 수 있고, 지자체 혹은 의료기관에서도 자체적으로 가지고 있을 수 있다.

관리지원체계와 명령대응체계의 두 가지 개념은 미국 질병통제예방센터(Centers for Disease Control and Prevention 이하 CDC)에서 표준화한 국가재난관리시스템(National Incident Management System)에 등장한다. 감염병으로 인한 공중보건 위기대응과 관련하여 연방(Federal)정부, 주(State)정부 및 지방(City and county)정부 등 모든 수준의 정부와 공공, 민간 및 비영리단체와의 조정을 원활히 하기 위해 '사건관리체계(Incident Management System)'와

'사건명령체계(Incident Command System)'를 갖추고 있다 [11]. 사건관리체계는 잠재적으로 공중보건의 위기를 초래하는 요인을 생물학적, 인위적, 의도적, 자연발생적으로 유형화하여 모든 가능한 공중보건 위협요소의 심각성, 발생가능성, 대응력을 기준으로 위험관리 순서를 정하여 대응하는 것이다 [12]. 미국 CDC가 주정부와 보건인적 서비스부(Department of Health and Human Service)를 지원한다. 사건명령체계는 발생시점을 기준으로 재정, 인력, 시설, 장비 및 위기 소통을 통제할 수 있는 임시 관리절차를 말한다. 보건인적 서비스부 장관이 명령센터(HHS Secretary's Command Center)를 구성하고 모든 보건의료 차원의 준비, 대응, 회복 활동을 수행한다. 하지만 사건 현장에서는 사건의 종류 및 발생 정도에 따라 사건 관리자(Incident manager)를 정하여 유연하게 대응할 수 있는 비상조직을 운영하며, 사건 대응에 참여하는 관련자들은 오로지 사건 관리자에게만 보고한다.

명령대응체계와 관리지원체계의 중요성은 거버넌스 문제로 환원될 수 있다. 2015년 5월 20일 국내에 첫 번째 메르스 환자가 확진 된 후 감염병 위기단계는 '주의'단계였다. 그럼에도 불구하고 비상조직의 형태는 질병관리본부(대책본부)에서 보건복지부(중앙 메르스 관리대책본부)와 국민안전처(범정부 메르스 대책지원본부) 그리고 청와대(메르스 긴급대책반)로 계속하여 상향 조정되었다. 6월 4일에는 '메르스 민관 종합대응 태스크포스'가 지휘하였고, 6월 8일에는 민간전문가 위주로 병원감염 관리 지도에 관한 전권과 행정지원 요청 명령권이 부여된 '즉각 대응TF'가 발족되었다. 6월 9일에는 '메르스 즉각 대응팀'까지 꾸려졌다. 명령의 지휘권도 청와대 대변인에서 총리대행, 정책조정 수석으로 바뀌었다가 마침내 대통령에 이르게 되는 등 명령대

응체계에 문제점을 드러냈다 [13].

관리지원체계는 매뉴얼로 구현될 수 있는데, 메르스 발생 후 매뉴얼이 서로 일치하지 않아 혼란이 가중되었다고 할 수 있다. 매뉴얼은 사전에 감염병의 위해 요소를 평가하여 작성되지만, 감염병 발생 후 특정한 행동을 확정하거나 필수적인 조치만을 선택해서 추가할 수도 있다. 하지만 매뉴얼이 바뀌더라도 혼란 없이 적용되도록 하는 거버넌스 역할은 무엇보다 중요하다.

B. 감염병의 예방 및 관리에 관한 법률(법률 제14316호)

1. 메르스 발생 후 입법경과

사우디아라비아에 이어 세계에서 두 번째로 큰 규모로 국내에서 유행했던 메르스는 확진 환자가 발생할 당시에는 법정 감염병로서 법적 지위를 가질 수 없었다. 법률 제2조에서 제4군 감염병은 보건복지부령인 시행규칙에 위임해 두었지만 메르스는 이 시행규칙에 포함되지 않았었고, 단지 법정 감염병 진단·신고기준에만 신종 감염병 증후군으로 명시되어 있었다. 그 후 개정을 거쳐 국내에서 새롭게 발생하였거나 발생할 우려가 있는 감염병 또는 국내 유입이 우려되는 해외 유행 감염병으로서 중동호흡기증후군(MERS)이라는 명칭으로 보건복지부 장관이 지정하는 제4군 감염병이 되었다.

감사원의 메르스 예방 및 대응 실태조사의 결과보고서에서 지적하는 초기대응 문제점은 최초 환자 신고가 있었으나 역학조사가 지체되었고, 의료기관 내에서 2차 감염 가능성을 검토하

지 않고 역학조사를 종료하였다는 것이다 [14]. 또한 의료기관의 문제점으로 대형병원 응급실의 과밀화와 다인실(多人室)처럼 운영되는 응급실 환경, 가족 간병으로 인한 감염 가능성의 증가 등이 문제로 파악되었다 [15]. 이에 따라 방역체계 개편과 감염병 전문 인력 보강, 병원 내 감염 관리감독 방안 마련, 응급실 구조 및 응급실 문화 개선, 의료기관 및 약국 등에 대한 손실보상, 환자 및 사망자에 대한 보상, 감염병으로 인한 공중보건 위기 시 정보요청과 정보공개를 골자로 감염병의 관리와 예방에 관한 법률이 개정되었다. 하지만 발생 초기 대응에 중요한 의료기관과 보건소간의 협력을 위한 거버넌스 문제, 충분한 입원 격리시설 등 병원단계의 대응 문제, 법정 감염병의 분류체계에 대한 논의는 계속되고 있다 [16].

2. 감염병의 예방 및 관리에 관한 법률의 문제점

A. 정책 및 관리측면

감염병의 예방과 관리는 그 법적 대상과 범위가 명확할수록 투명한 의사결정과 신속한 대응 조치가 가능하다. 그러나 공중보건 위해 요소가 무작위적이고 비정형적인 형태로 증가하고 있어 그 영향력과 심각도를 정확히 평가하여 감염병을 분류하고 그 범위를 설정하는 일은 점점 더 어려워지고 있다. 발생률과 사망률, 역학적 정보를 바탕으로 질병발생 근거, 지역에서 발생 가능성이나 혹은 해외로부터 유입 가능성, 예방 및 치료 가능성, 대중들의 질병에 대한 민감도 등을 기준 삼아 평가하는 것 이외에도 감염병 분류를 위한 평가기준은 다양하다.

미국 CDC에 의해 개발된 인플루엔자 위험평가 도구(Influenza Risk Assessment Tool)는 인플루엔자 바이러스에 의해 야기 될 잠재적인 위험을 평가하는 바이러스 특성과 인구집단의 특성 그리고 생태학 및 역학적 기준을 제시한다 [17]. 유럽연합은 매년 계절인플루엔자 위험평가를 위해 임상적·역학적·바이러스학적 데이터뿐만 아니라 인플루엔자의 영향을 받은 나라에서 설문조사를 시행하여 감염병의 심각성을 평가한다 [18]. 세계 보건기구의 인플루엔자 대유행 평가도구(Tool for Influenza Pandemic Risk Assessment)는 영향력, 확산 위험성, 여행이나 무역 중단의 필요성을 평가기준으로 삼고 있다 [19].

감염병의 분류는 신고체계와 연관된다. 감염병마다 보건소와 질병관리본부에 신고·보고하는 시기가 상이하기 때문이다. 감사원 자료에 따르면, 2015년 10월-2016년 9월 동안 수두를 병명으로 요양급여를 청구한 서울시 1,499개 의료기관을 조사한 결과 81.5%인 1,221개 기관이 수두 발병을 제대로 신고하지 않았고, 유행성이하선염의 경우에도 표본 조사한 824개 의료기관 가운데 656개 기관인 79.6%가 제대로 신고하지 않았다 [20].

법 제2조 8-12항은 보건복지부 장관이 고시하는 감염병을 법적 대상으로 하고 있어 감염병을 일반적·추상적 성격인 행정처분으로 이해하게 됨으로써 신속한 진단과 신고가 제때에 이루어지지 않을 개연성이 높다 [21]. 고시의 법적 성질은 행정기관이 불특정 다수인에게 통보하는 행정처분으로서 단지 어떤 사실을 널리 알리는 기능만을 할 뿐이고, 그 자체만으로는 국민의 권리의무와 관련하여 법적 효과를 가져 오는 것은 아니다. 고시가 법규명령으로서 성질을 갖추기 위해서는, 구체적이고 명확한 법률의 위임을 요구한다 [22]. 위임의 근거가 되는

Volume: 39, Article ID: e2017033

https://doi.org/10.4178/epih.e2017033

해당 개별조항을 법률에 구체적으로 명시할 것을 요구하는 것은 국민의 기본권을 보호하기 위한 것이다. 국민의 기본권 보호라는 국가 의무와 이동의 자유와 같은 개인의 권리간 충돌이 있을 수 있는 감염병 대응 조치의 특성에 비추어 볼 때 고시는 기본권 보호 차원에서 한계가 있다. 또한 구체적 규범통제로서 고시의 효력은 다른 법령 및 공고 문서에 특별한 규정이 있는 경우를 제외하고는 고시가 있은 후 5일이 경과한 날부터 발생한다 [23]. 이는 격리와 검역 등 현장에서 이루어질 수 있는 즉시 강제조항과 모순되는 형식이라고 할 수 있다.

B. 의료자원의 조직과 보건의료서비스 제공측면

가장 먼저 감염병 대응 조치를 하는 사람은 의심환자를 신고하는 의료인이라고 할 수 있다. 의료인의 책무는 소속의료기관의 장에게 보고하거나 혹은 의료기관에 소속되지 아니할 경우에는 관할 보건소장에게 신고하는 것이다(법 제11조). 법 제18조에 따르면, 의료인이나 의료기관장이 보건복지부 장관 등에 역학조사 실시를 요청할 수 있다. 역학조사 요청의 목적은 질병이 발생했거나 발생할 우려가 있다는 사실을 알게 된 경우 역학조사가 신속하게 수행되기 위한 것임에도 불구하고 이 법 조항은 환자를 진료한 의료인 등만 역학조사 실시를 요청하는 것으로 축소 해석될 수 있다. 만약 역학조사를 요청할 수 있는 자격을 한정할 필요성이 인정된다면, '감염병 등이 발생하였거나 발생할 것이 우려되는 환자를 진료한 의료인' 등으로 개정이 필요하다.

법 제42조는 감염병에 관한 강제처분 조항으로서 권한의 주체는 보건복지부 장관, 시·도

Volume: 39, Article ID: e2017033

https://doi.org/10.4178/epih.e2017033

지사 또는 시장·군수·구청장이다. 감염병 환자 등이 있다고 인정되는 현장에서 조사하고 진찰하거나 치료받게 하거나 입원시킬 수 있는 강제권한이지만 병렬로 명시되어 있으니 우선 결정권자가 누구인지 혼선을 준다. 시·도와 보건소의 역학조사관은 동일한 권한을 가지고 현장에 출동할 수 있으므로 현장에서의 혼란은 불가피해 보인다. 법 제47조에 의하면, 감염병 발생 시 해당 장소를 일시적으로 폐쇄 조치할 수 있는 권한은 역학조사관이 갖고 있다. 긴급한 경우 역학조사관에게 감염병 관리를 위한 잠정조치를 취할 수 있도록 하려는 취지는 인정되나, 해당 장소의 일시적 폐쇄 조치 권한을 가지고 있다는 것이 어떤 행정활동인지 모호하다. 이와 유사한 현장 지휘권은 법 제60조에 따라 방역관이 가지고 있다. 방역관은 시·도의 소속 공무원 중에서 임명되기도 하고 보건복지부 소속 공무원이 될 수도 있다. 이들은 감염병 발생 현장에서 대응 조치 권한을 갖고 있는데, 이들의 업무와 현장에서의 역학조사관의 업무가 충돌할 수 있다. 어떠한 경우에 어떠한 조치를 할 수 있는지 방역관과 역학조사관의 직무범위와 그 내용이 법률에 상향 규정됨이 바람직할 것이다.

감염병 발생현장에서 대응조치가 재난관리 활동의 하나라면 재난 및 안전관리 기본법과의 정합성도 갖추어야 한다. '재난 및 안전관리 기본법' 제30조에 따른 긴급 안전점검 업무에 종사하는 4급부터 9급까지의 국가공무원 및 지방공무원들의 업무는 '사법경찰 관리의 직무를 수행할 자와 그 직무범위에 관한 법률'에 명시되어 있다. 동 법률에는 특별시·광역시·특별자치시·도·특별자치도 및 시·군·구에 근무하는 공무원의 역할이 명시되어 있다. 방역관의 현장 지휘권도 공무원이 행사하는 강제권한이므로 이 업무범위에 공중보건 대응 조치 업무가 있어

야 할 것이다.

C. 자원조직화와 재정지원측면

감염병 위기경보단계에 따라 조직되는 비상조직의 방역대책과 업무내용은 감염병의 위험도에 따라 상이하다. 고위험 감염병은 질병관리본부가 총괄하여 지휘한다. 시·도와 시·군·구는 위험도가 낮은 감염병 발생에 대응한다. 그래서 주관 기관과 주체가 감염병 발생현장, 실험·진단실, 지정치료 및 격리의료기관, 검역·격리장소에 따라 상이할 수 있다. 그러므로 국가개입의 필요성 정도가 감염병의 위험도와 비례되도록 비상조직 구성을 위한 보건행정에 대한 정의(定義)와 범위가 명시되어야 하고, 권한기관과 명령권자가 병기되어야 한다. 법률 조항에 명시되어 있지 않다면 공중보건 실무를 담당하는 공무원들의 역무가 가장 밀접한 것을 중심으로 서로 덜 간섭하게끔 조정해주는 거버넌스가 필요하다. 예컨대 격리입원조치를 위해서는 검역소가 질병관리본부에 요청하는 경우와 보건소가 시·도에 요청하는 경우 국가지정 입원치료병상이 배정된다. 이 과정에서 배정의 우선순위 문제가 발생할 경우 이를 조정해주는 관리체계가 있어야 할 것이다.

긴급 의약품의 사용은 의약품 분야의 자유무역협정(Free Trade Agreement)체결에 따른 의약품의 허가·심사·상호인증이 필요하므로 각국의 허가심사체계에 대한 이해를 바탕으로 국제기준에 부합되도록 의약품의 품질, 비 임상시험, 임상시험 기준 등을 국내 법률에 반영해야 한다[24]. 외국에서 개발된 의약품을 국내로 도입할 때 요구하는 자료 요건의 차이로 인

하여 품목허가가 지연되거나 거부되는 경우가 발생할 수 있기 때문이다.

미국의 경우, '감염병 대유행 및 재난대비 법(Pandemic and All-Hazards Preparedness Act 2006)'을 제정하여 법체계를 완성하였고, FDA의 미승인 의약품에 대한 긴급 사용권이 강화되었다 [25]. '감염병 대유행 및 재난대비 재승인 법(Pandemic and All-Hazards Preparedness Reauthorization Act 2013)'은 비상시 승인되지 않은 의약품의 용도에 대한 비상 사용권을 규율 한다 [26]. '21세기 치유법(21st Century Cures Act)'은 FDA 허가 승인에 요구되는 임상 근거의 폭을 확대하고, 일부 사례의 경우 임상연구 요건을 면제하여 새로운 의약품 및 의료기기 개발의 촉진과 신속한 허가를 도모하기 위해 2015년 미국 하원의 해당 위원회에서 만장일치로 가결되었다 [27]. 하지만 이러한 법률은 현행 기준이 허용하는 안전성·유효성보다 열등한 의약품 및 의료기기가 허가나 승인될 가능성이 있다 [28]. 그러므로 신속한 허가·승인 절차 구축에 있어 중요한 것은 환자와 보건의료체계에 좋지 않은 결과가 야기되지 않도록 신속평가기준을 마련하는 것이다.

IV. 논의

감염병의 발생요건은 늘 변화하여 좀처럼 조기에 인식하기 어렵고, 복잡하며 때로는 모순되기도 한다. 그래서 감염병의 대비와 대응 조치는 국가가 단독으로 해결할 수 없는 '고약하고도 난해

한 문제'라고 할 수 있다 [29]. 점점 더 다양해지는 원인불명의 신종 감염병과 의도적 테러, 도시의 각종 대형사고 등 다양한 공중보건 위협에 대응하기 위한 법률은 인권을 침해하지 않는 한계 내에서 국가 개입이 이루어질 수 있는 안정된 근거가 되어야 한다. 모든 국민은 존중 받아야 할 존엄성이 있고, 그 존엄성은 모든 기본권 주체에게 보편적으로 보장됨으로써 공동의 이익이 되어야 하기 때문이다.

우리나라는 '제3차 국민건강증진종합계획 2011~2020'을 수립하여 HIV/AIDS에 대한 사회적 편견과 차별을 개선하기 위해서 에이즈 환자에 대한 차별적 태도 수준을 2008년 44.6%에서 2020년에는 15.0%로 개선하는 것을 목표로 하는 정책을 시행하고 있다 [30]. 헌법이 보장하고 있는 권리에 초점을 맞추는 것뿐만 아니라 인권을 인간의 존엄성과 보편적인 가치로 이해하여 감염병의 관리와 대응을 위해 필요한 법률과 거버넌스가 진지하게 모색되기를 기대하며 다음과 같이 제언한다.

A. 감염병 관련 법의 개정방향

감염병의 예방 및 관리에 관한 법률은 역학조사, 검역과 격리, 실험실 진단, 예방접종과 치료의 근거가 되는 특별법 역할을 한다. 동시에 감염병 발생을 사회재난으로 관리할 수 있도록 기본법 역할도 해야 한다. 감염병 관련 법률이 감염병 관리와 대응 조치의 특수성을 반영하되 국가와 지자체의 개입을 통해 헌법 제34조, 제35조, 제36조에 따른 국민의 생명, 신체, 건강과 재산을 보호하는 의무를 수행하는 기본법이 되어야 할 것이다.

Volume: 39, Article ID: e2017033

https://doi.org/10.4178/epih.e2017033

1. 거버넌스

신종 감염병의 관리를 위한 거버넌스의 형성은 국제법적 전략(international legal strategy) [31]이 필요한 만큼 그 적용범위는 IHR 2005의 주요 변화를 반영하는 차원에서 이루어져야 한다. 국내 메르스 발생은 지역적인 감염병으로 시작했지만 국가재난으로 간주되어 중앙재난안전대책본부가 구성되었다. 이는 신종 감염병의 발생 초기 현장대응단계의 중요성을 일깨워 주는 사례이며, 지자체의 권한과 실행력을 통해 보다 신속하고 효과적인 재난관리가 이루어질 수 있었을 것이라는 아쉬움이 남는 부분이다. 발생 지역에서 심각한 상황으로 판단되면, 가장 근거리에 위치한 지자체가 먼저 위기상황에 맞는 현장 대응이 가능하도록 실질적 법적 지위를 갖고 이에 합당한 기능을 수행할 수 있는 체계를 갖추어야 놓아야 한다.

지자체의 재난관리체계, 유관기관 간의 협조체계, 현장지휘체계가 법률에 명시되어야 한다. 재난관리 주관기관은 중앙행정기관이지만 재난관리 책임기관은 지자체가 될 수 있다. 사무의 성질에 따라 그에 대한 경비부담과 최종적인 책임귀속의 주체가 달라질 수 있다. 지금은 재난 유형에 따라 책임기관을 다르게 규정하고 있지만 향후에는 광역자치단체와 기초자치단체에 따라 책임의 범위를 구분하는 것이 바람직하다. 또한 지방자치단체의 장이 처리하도록 규정하고 있는 사무가 기관위임사무에 해당하는지 여부를 판단하고 체계적으로 협력하기 위해서 지자체의 조례를 마련해야 한다. 그리고 감염병으로 인한 위기상황과 다른 위협요소로 야기된 위기상황을 구분하여 자치구간의 상호지원과 행정응원을 요청하거나 명령할 수 있도록 위임조항이

Volume: 39, Article ID: e2017033

https://doi.org/10.4178/epih.e2017033

필요하다.

공중보건위기 시 구성되는 비상조직은 위기단계에 따라 상이하며, 관련 지자체의 대응 조치 내용은 달라질 수 있다. 법률에서는 그 내용이 자세히 명시되어 있지 않고, 예외적인 상황에 따른 역할분담은 매뉴얼에 선택적으로 명시하고 있다. '감염병 현장조치 행동매뉴얼'은 재난 및 안전관리 기본법을 근거로 재난의 예방•대비•대응•복구와 그밖에 필요한 사항을 담아 시장•군수•구청장이 작성하도록 되어 있다. 문제는 두 개의 광역자치단체 이상의 협력을 요구하기 위해서는 사회재난으로 판단될 정도로 그 피해범위가 심각해야 하는데, 만약 여러 광역자치단체에 걸쳐 피해에 발생하지 않으면 협력이 이루어지지 않을 수 있다. 그러므로 발생범위를 기준으로 협력 규모를 조정하여 매뉴얼을 정합성 있게 정리하여야 한다.

2. 긴급치료

허가받지 않거나 신고하지 않은 의약품을 긴급사용하기 위해서는 적어도 세 가지 범위의 의무사항이 법률에 명시되어야 할 것이다. 긴급사용 승인의약품의 환자안전관리대책 마련 의무, 의약품의 신속사용을 위한 표준절차 개발의무, 그리고 공중보건 위기상황 별 의약품 신속사용 결정을 위한 협의의무다.

긴급사용 승인 의약품의 환자안전관리대책을 위해서 응급사용 허가제도가 마련되어야 한다. 해당 의약품이 이전에 허가를 받은 적응증과는 다른 용도로 사용하는 경우와 이전에 허가받은 적이 없는 의약품의 경우로 나누어 구체적인 승인절차를 마련해야 한다. 현재 약사법•의료기기

Volume: 39, Article ID: e2017033

https://doi.org/10.4178/epih.e2017033

법 상 의약품·의료기기의 안전성·유효성 등을 검증하기 위해서는 시판 전에 품목허가 또는 신고를 받아야 한다 [32]. 이러한 과정 없이 신속하게 평가하고 사용을 허가하기 위해서는 일반적인 승인절차와는 다른 별도의 절차가 필요하다. 미국의 경우, 동물실험 결과에 근거하여 사람에게도 임상적으로 유익할 것으로 유추하여 의약품을 허가할 수 있도록 하는 '애니멀 룰(Animal Rule)'을 적용하고 있다 [33]. 애니멀 룰에 의하면 긴급사용 승인을 받기 위한 제출자료 목록 개발의 중요하다. 대상 의약품의 사용목적, 의료적 필요성, 허가 상태, 안전성·유효성 정보, 제조와 품질정보, 위험성·유익성 평가자료, 의약품 관련 사실 설명서 등 긴급사용 승인을 받기 위한 검토자료의 목록 개발이 필요하다. 그리고 긴급 사용승인 의약품 등의 사용기간·범위·조건 등 그밖에 필요한 사항을 보건복지부 장관이 정할 수 있도록 하려면 신종 감염병 발생 전 단계부터 질병관리본부와 식품의약품안전처는 이러한 정보를 공유하고 협의해야 할 것이다.

3. 인권보장

A. 역학조사 대상자의 범위 개선

감염병의 유행기간 동안 공무원에 의한 강제처분의 정당성을 확보하기 위해 최소침해의 원칙과 비례의 원칙이 실효를 거둘 수 있도록 강제조치의 대상 및 방법과 그 수행절차의 개선이 필요하다 [34]. 기본권 제한으로 추구되는 이익이나 기본권 제한으로 상실되는 이익이나 모두 기본권 주체의 보편적이고 본질적인 가치이므로 이를 보장하는 형식으로 추구되어

야 하기 때문이다.

역학조사 대상자의 분명한 구분은 최소침해의 원칙에 의한 국가 개입의 한계와 연관된다. 법 18조 제3항에서 규정하는 역학조사 대상자는 '누구든지'이다. 그러나 법 제41조에 근거한 역학조사 대상자는 감염병 환자 등이다. 감염병 환자 등은 의학적인 관점에서 '감염병 환자, 감염병 의사환자, 병원체 보유자를 말한다. 하지만 이들에 대한 조치에 있어서는 모두 동일한 행정명령의 대상으로 간주하여 국민의 기본권 측면에서는 무심하다.

역학조사의 목적이 각 확진 환자가 어떻게 감염되었는지 확인 또는 추정하고 각 확진 환자와 밀접하게 접촉 한자가 추가 환자로 확진될 수 있는지 그 가능성을 평가하는 것이라면, 역학조사 대상자는 감염병에 감염될 위험성을 기준으로 고위험군, 중위험군, 저위험군, 무위험군으로 분류할 수 있을 것이다. 이렇게 분류된 각 집단은 다시 증상군과 무증상군으로 나눌 수 있다[35]. 특히 무증상군에 대한 검역과 격리조치는 자유 행사의 범위를 제한하는 것이므로 과학적인 근거에 의해 그 자유 제한 범위와 조건을 제시해 주어야 할 것이다.

법 제42조는 감염병 환자 등에 대한 강제처분 관련 조항이다. 특정한 감염병에 해당하는 감염병 환자 등에 대하여는 강제조사 및 진찰을 할 수 있고 그 진찰 결과, 감염병 환자 등으로 인정될 때에는 강제입원을 시킬 수 있다. 감염병 환자 등에 대한 강제처분은 제41조의 입원 치료 의무와는 차이가 있다. 강제 진찰이나 강제 입원조치는 행정상 즉시 강제의 일종이다. 이는 '행정상 상해가 발생하거나 장애의 발생이 목전에 급박한 경우, 개인에게 의무를 명해서는 행정목적을 달성할 수 없거나 또는 미리 의무를 명할 시간적 여유가 없는 경우'에

Volume: 39, Article ID: e2017033

https://doi.org/10.4178/epih.e2017033

시행하는 것이다 [36]. 행정상 즉시 강제의 정당성을 인정받기 위해서는 국민의 기본권을 침해할 소지가 크다는 점에 유념하여 그 도입에 신중해야 하며 즉시 강제보다 기본권 침해 정도가 덜하다고 인식되는 방법을 우선 사용해야 한다 [37]. 그러므로 강제 조치의 구체적인 조건과 절차, 격리입원 기간, 퇴원조건, 의견진술 기회 등에 대한 구체적인 규정이 마련되어야 할 것이다 [38].

B. 후천성 면역결핍증 예방법과 결핵예방법의 개선

감염병의 예방 및 관리에 관한 법률은 '전염병예방법(법률 제308호)'을 2009년에 전면 개정한 것으로서 전염병이라는 명칭을 더 이상 사용하지 않는다. 그리고 같은 해에 기생충질환 예방법이 폐지되었다. 하지만 1967년 1월과 1987년 11월에 각각 제정된 결핵예방법과 후천성면역결핍증 예방법은 그대로 존속되고 있다 [39]. 이렇게 질병명으로 된 별도의 법률로 규율하는 것이 해당 감염병 환자에게 지속적이고 중대한 인권의 제한을 강요하는 것은 아닌지 실증적인 검토가 필요하다.

우선 감염병의 예방 및 관리에 관한 법률과 이 두 가지 법률 사이에 의무나 처벌의 내용을 달리할 합리적인 이유가 있는지 살펴보아야 한다. 감염병의 예방 및 관리에 관한 법률은 역학조사 대상자의 거부행위 즉, 거짓 진술 또는 거짓 자료 제출행위와 고의적인 사실 누락·은폐 행위의 경우, 일률적으로 '2년 이하의 징역 또는 2천만 원 이하의 벌금'에 처한다(법 제79조). 하지만 감염병 환자 등의 입원조치나 자택격리조치 위반에 대해서는 300만 원 이하의

벌금을 부과하고, 재난 시 의료인에 대한 거짓 진술의 금지(법 제35조) 위반에 대하여는 1,000만 원 이하의 과태료를 부과하고 있다. 후천성 면역결핍증 예방법에서는 전파매개행위를 한 사람에 대하여는 3년 이하의 징역에 처하도록 하고(법 제25조), 1년 이하의 징역 또는 300만 원 이하의 벌금을 부과하고 있다(법 제10조 및 제27조 제2호). 결핵예방법은 처벌규정이 없다 [40].

역학조사의 중요성을 강조한다면, 역학조사의 거부로 인한 감염병 확산에 대한 처벌규정과 처벌하지 않는 단순 역학조사 거부행위에 대한 규정으로 이원화하는 것이 타당할 것이다. 역학조사에 대한 협조가 이루어지지 않아 질병 확산의 급박한 위험요인이 되고, 손해 발생의 개연성까지 있어서 책임을 물을 수 있는 경우가 있다. 반면 무증상 의심환자와 같이 강제 격리조치가 없다고 해도 공중보건에 위해가 발생할 수 있을만한 명백하고 확실한 증거가 없는 경우도 있기 때문이다. 의무이행 대상자를 이원화하는 것이 의학적으로 타당하다면, 벌칙조항을 형평성 있도록 개선하는 것이 정당할 것이다.

이원화가 타당하지 않다면, 역학조사 대상자의 범위를 통일해야 한다. 후천성 면역결핍증 예방법과 결핵예방법에서는 역학조사 대상을 '감염인 및 감염이 의심되는 충분한 사유가 있는 사람'이라고 정의하고 있다. '충분한 사유'가 의미하는 바에 대한 구체적인 설명도 없이 이 두 감염병에 대해서만 별도의 집행 작용이 필요한 것으로 보고, 협력 의무자를 달리 정의한 것이라면, 법익의 균형성과 수단의 적합성 원칙에 위배된다고 할 수 있다. 기본권의 이익은 보편적인 이익이고, 보편적 기본권을 제한하는 사유는 그 대상에 상응하여 동일한 정당화에

Volume: 39, Article ID: e2017033

https://doi.org/10.4178/epih.e2017033

기초하여야 한다 [41].

Volume: 39, Article ID: e2017033

https://doi.org/10.4178/epih.e2017033

REFERENCES

1. Fidler DP. Globalization, international law, and emerging infectious diseases. Emerg Infect Dis 1996;2:77-84.

2. Kleczkowski BD, Roemer MI, Van Der Werff A. National health systems and their reorientation towards health for all: guidelines for policy-making; 1984 [cited 2017 Aug 28]. Available from: http: //apps.who.int/iris/bitstream/10665/41638/1/WHO_PHP_77.pdf.

3. Ministry of Health and Welfare. Basic plan for prevention and management of infectious diseases (2013-2017) [cited 2017 Aug 28]. Available from: http://www.mohw.go.kr/front_new/jb/sjb-030301vw.jsp?PAR_MENU_ID= 03&MENU_ID= 0319&CONT_SEQ= 293140&page= 1 (Korean).

4. Choi JW, Lee JS, Kim KH, Kang CH, Yum HK, Kim Y, et al. Proposed master plan for reform of the national infectious disease prevention and management system in Korea. J Korean Med Assoc 2015;58:723-728 (Korean).

5. Coker R. Mounier-Jack S. Pandemic influenza preparedness in the Asia–Pacific region. Lancet 2006;368:886-889.

6. Gostin LO. World health law: toward a new conception of global health governance for the 21st century. Yale J Health Policy Law Ethics 2005;5:413-424.

7. World Health Organization. International health regulations (2005): areas of work for implementation; 2007 [cited 2017 Aug 28]. Available from: http://www.who.int/ihr/finalversion9Nov07.pdf.

8. Choi BY. Overview of emerging infectious diseases in Korea. Korean J Epidemiol 2008;30:147-155 (Korean).

9. Turnock BJ. Public health: what it is and how it works. 5th ed. Burlington: Jones & Bartlett Learning; 2012, p. 160-194.

10. World Health Organization. Strengthening health leadership and management: the WHO framework [cited 2017 Aug 28]. http://
www.who.int/workforcealliance/forum/SBW_WHO_Leadership_and_Management_Framework.pdf?ua

Volume: 39, Article ID: e2017033

https://doi.org/10.4178/epih.e2017033

=1

11. Annelli JF. The national incident management system: a multiagency approach to emergency response in the United States of America. Rev Sci Tech 2006;25:223-231.
12. New York City Department of Emergency Management. New York City community emergency response teams standard operation procedures; 2015 [cited 2017 Aug 28]. Available from: http://www.nyc.gov/html/oem/downloads/pdf/cert/cert_web_sops.pdf.
13. Ministry of Health and Welfare. The 2015 MERS outbreak in the Republic of Korea: learning from MERS; 2015 [cited 2017 Aug 28]. Available from: https://www.mohw.go.kr/front_new/jb/sjb-030301vw.jsp?PAR_MENU_ID = 03&MENU_ID = 032901&CONT_SEQ= 337407& page= 1 (Korean).
14. Board of Audit and Inspection of Korea. MERS prevention and response; 2015 [cited 2017 Sep 4]. Available from: http://www.bai.go.kr/bai/search/search.do?kwd= MERS&category= categor y6&subCategory= 22&reSrchFlag= false&pageNum= 1&pageSize= 10&detailSearch= false&srchFd= all&sort= r&date= null&startDate= &endDate= &writer= &preKwd= MERS (Korean).
15. World Health Organization. Managing contacts in the MERSCoV outbreak in the Republic of Korea; 2015 [cited 2017 May 10]. Available from: http://www.who.int/mediacentre/news/mers/ briefing-notes/update-1-july-2015/en/.
16. Choi MR. Infectious disease classification system reform. Health Focus; 2016 Dec 12 [cited 2017 May 10]. Available from: http://www.healthfocus.co.kr/news/articleView.html?idxno= 66588&sc_word= mil0726 (Korean).
17. Centers for Disease Control and Prevention. Influenza Risk Assessment Tool (IRAT) [cited 2017 May 10]. Available from: https://www.cdc.gov/flu/pandemic-resources/national-strategy/riskassessment.htm.
18. European Centre for Disease Prevention and Control. Why do I need a flu vaccine every year? [cited 2017 May 10]. Available from: https://ecdc.europa.eu/en/publications-data/why-do-i-need-fluvaccine-every-year.
19. World Health Organization. Tool for influenza pandemic risk assessment; 2016 [cited 2017 Aug 28]. Available from: http://apps.who.int/iris/bitstream/10665/250130/1/WHO-OHE-PED-GIP-

2016.2-eng.pdf.

20. Board of Audit and Inspection of Korea. Medical care claim: chickenpox outbreak; 2017 [cited 2017 May 1]. Available from: http://www.bai.go.kr/bai/search/search.do?kwd= %EC%9A%94%EC%96%91%EA%B8%89%EC%97%AC%EC%B2%AD%EA%B5%AC.+%EC%88%98%EB%91%90%EB%B0%9C%EB%B3%91&category= all&preKwd= %EB%A9%94%EB%A5%B4%EC%8A%A4+%EC%98%88%EB%B0%A9+%EB%B0%8F+%EB%8C%80%EC%9D%91%EC%8B%A4%ED%83%9C&x= 0&y= 0 (Korean).

21. Park MJ, Lee JG. A study on legal problems and proposal for an enactment regarding MERS in the Republic of Korea. Korean J Med Law 2015;23:191-209 (Korean).

22. Song DS. Rechtsnatur der behördlichen Bekanntgaben(Gosi) und Rechtsschutz. Public Land Law Rev 2011;52:239-267 (Korean).

23. National Law Information Center. Regulations Governing Efficient Management of Administrative Affairs No.18746 [cited 2017 May 10]. Available from: http://law.go.kr/lsInfoP.do?lsiSeq= 67169#0000 (Korean).

24. Jung YR, Lee JH, Choi JY, Cheong JH, Chun IK. Comparative study on ICH quality guidelines and their related KFDA regulations. Regul Res Food Drug Cosmet 2011;6:107-117 (Korean).

25. US Department of Health and Human Services. Pandemic and All Hazards Preparedness Act [cited 2017 May 10]. Available from: http://www.phe.gov/preparedness/legal/pahpa/pages/default.aspx.

26. US Department of Health and Human Services. Pandemic and All Hazards Preparedness Reauthorization Act of 2013 (PAHPRA) [cited 2017 May 10]. Available from: http://www.fda.gov/EmergencyPreparedness/Counterterrorism/MedicalCountermeasures/MCMLegalRegulatoryandPolicyFramework/ucm359581.htm.

27. Committee on Energy and Commerce. Rules committee print 114-22, text of H.R. 6, 21st Century Cures Act; 2015 [cited 2017 Aug 28]. Available from: https://www.gpo.gov/fdsys/pkg/CPRT-114HPRT95335/pdf/CPRT-114HPRT95335.pdf.

28. Avorn J, Kesselheim AS. The 21st Century Cures Act: will it takeus back in time? N Engl J Med 2015;372:2473-2475.

29. Rittel HWJ, Webber MM. Dilemmas in a general theory of planning. Policy Sci 1973;4:155-169.
30. Lee CH. Criminal policy for infectious disease control and prevention. Seoul: Korean Institute of Criminology; 2015, p.1-162 (Korean).
31. World Health Organization. WHO guidance for the use of Annex 2 of the International Health Regulations (2005); 2010 [cited 2017 July 7]. Available from: http://www.who.int/ihr/publications/annex_2_guidance/en/.
32. National Law Information Center. Enforcement Decree of the Pharmaceutical Affairs Act, No.27673; 2016 [cited 2017 May 10]. Available from: http://www.law.go.kr/eng/engLsSc.do?menuId=1&query= Enforcement+Decree+of+the+Pharmaceutical+Affairs+Act&x= 0&y= 0#liBgcolor0 (Korean).
33. US Department of Health and Human Services; Food and Drug Administration. Product development under the animal rule guidance for industry; 2015 [cited 2017 Aug 28]. Available from: https://www.fda.gov/downloads/drugs/guidances/ucm399217.pdf.
34. Park DG, Park CG, Song CH, Oh JH. Disaster response in local governments. Seoul: Daeyongmunhwasa; 2009, p. 146-189 (Korean).
35. Bialek SR, Allen D, Alvarado-Ramy F, Arthur R, Balajee A, Bell D, et al. First confirmed cases of Middle East respiratory syndrome coronavirus (MERS-CoV) infection in the United States, updated information on the epidemiology of MERS-CoV infection, and guidance for the public, clinicians, and public health authorities -May 2014. MMWR Morb Mortal Wkly Rep 2014;63:431-436.
36. Hong JS. Administrative law principles. 1st ed. Seoul: Parkyoungsa; 2014, p. 481-540 (Korean).
37. Lee JS. Research on constitutional issues concerning the protection of people's lives and bodies. Constitutional court of Korea; 2015 [cited 2017 May 10]. Available from: http://search.ccourt.go.kr/ths/pt/selectThsPt0101List.do (Korean).
38. Choung HU. Study on the administrative immediate enforcement. Kyungpook Natl Univ Law J 2012;40:275-300 (Korean).
39. Korea Centers for Disease Control and Prevention. Amendment of Tuberculosis Prevention Law; 2011 [cited 2017 Aug 28]. Available from: http://cdc.go.kr/CDC/info/CdcKrInfo0301.jsp?menuIds= HOME001-MNU1154-MNU0005-MNU0037&cid=12593 (Korean).

Volume: 39, Article ID: e2017033

https://doi.org/10.4178/epih.e2017033

40. Ministry of Health and Welfare. National Health Plan 2020; 2011 [cited 2017 May 10]. Available from: http://www.mohw.go.kr/front_new/jb/sjb030301vw.jsp?PAR_MENU_ID= 03&MENU_ID= 0319&CONT_SEQ= 257824&page= 1 (Korean).

41. Kymlicka W. Liberal individualism and liberal neutrality. Ethics 1989;99:883-905.
